# Coupled down-regulation of mTOR and telomerase activity during fluorouracil-induced apoptosis of hepatocarcinoma Cells

**DOI:** 10.1186/1471-2407-7-208

**Published:** 2007-11-12

**Authors:** Xinxin Bu, Fengqi Jia, Weifeng Wang, Xianling Guo, Mengchao Wu, Lixin Wei

**Affiliations:** 1 Tumor Immunology and Gene Therapy Center, Eastern Hepatobiliary Hospital, Second Military Medical Universisty, 225 Changhai Road, Shanghai 200438, China

## Abstract

**Background:**

Hepatocellular carcinoma (HCC) is the most invasive and frequently diagnosed malignancy and the second leading cause of cancer death in many regions of Asia. The PI3K/Akt/mTOR signal pathway is involved in multiple cellular functions including proliferation, differentiation, tumorigenesis, and apoptosis. Up-regulation of telomerase activity is thought to be a critical step leading to cell transformation.

**Methods:**

This study investigated changes in mTOR pathway and telomerase activity in hepatocarcinoma cell line SMMC-7721 treated with chemotherapeutic agent 5-fluorouracil (5-Fu). We detected apoptosis of hepatocarcinoma cells by TUNEL assay. Telomerase activity, hTERT transcription level and p- p70 S6k was demonstrated by the telomeric repeat amplification protocol and silver staining assay, Dual-Luciferase Reporter Assay and Western blot analysis respectively.

**Results:**

Treating SMMC-7721 cells with 5-Fu leads to apoptosis of the cells, and reduction in telomerase activity, as well as a dramatic reduction in the activated form of p70 S6 kinase, a mTOR substrate. The 5-Fu treatment nearly abolishes transcription of hTERT (the major component of telomerase) mRNA. Treating SMMC-7721 cells with Rapamycin, a specific mTOR inhibitor, significantly reduce hTERT protein level but did not affect hTERT transcription. 5-Fu and rapamycin were synergistic in regards to down-regulation of telomerase activity in hepatocarcinoma cells.

**Conclusion:**

These results suggest that chemotherapeutic agent 5-Fu may down-regulate telomerase activity at both transcriptional level and PI3K/Akt/mTOR pathway-dependent post-transcriptional level to facilitate hepatocellular carcinoma cell apoptosis.

## Background

Hepatocellular carcinoma (HCC) is the most invasive and frequently diagnosed malignancy and is the second leading cause of cancer death for men in China and some other parts of Asia [1]. Phosphatidylinositol-3-kinase (PI3-K) pathway has been reported as an important intracellular mediator frequently activated in cancer cells [2]. PI3K activates a number of signaling molecules, among which the Akt/mTOR pathway is of particular interest because of its role in inhibiting apoptosis and promoting cell proliferation [3]. The mammalian target of rapamycin, mTOR, also known as FRAP, RAFT1, or RAPT1, has been shown to regulate mitogen stimulated protein synthesis and cell cycle progression [4-6]. Cell culture studies have demonstrated that one of the mechanisms by which mTOR controls protein synthesis is through phosphorylating downstream substrates, including p70s6 kinase (p^70S6K1^) and eukaryotic initiation factor (eIF) 4E binding protein 1 (4E-BP1) [7-9]. The protein p^70S6K1 ^phosphorylates the 40S ribosomal protein S6 and is proposed to play a crucial role in the translation of 5'-terminal oligopyrimidine tract mRNAs, which primarily encode ribosomal proteins and components of the translation apparatus [10,11]. Phosphorylation by mTOR of 4E-BP1 disrupts its binding to eIF4E, a protein that binds the 5'-cap structure of mRNA. The released eIF4E allows the formation of a functional translation initiation complex containing eIF4G, eIF4A, eIF3, thereby allowing translation [12,13].

Rapamycin, an immunosuppressive macrocyclic lactone, specifically inhibits the activity of mTOR. Inhibition of mTOR leads to G1 arrest of many malignant cell lines, and currently analogs of rapamycin are being investigated as cancer therapeutic agents [14,15]. In many cell lines, exposure to rapamycin results in a relatively small decrease in overall protein synthesis (~15–20%), but dose result specifically in G1 cell cycle arrest. This can, in part, be explained by the fact that some cell cycle regulators, e.g. cyclin D1, c-MYC and growth factors such as IGF-α are controlled by the mTOR pathway [16-18]. Some studies also suggest that mTOR may be a cellular context-dependent, pleiotropic regulator of apoptosis, although conclusive demonstration of mTOR inactivation in such circumstances is lacking [19].

Telomerase is a specialized type of reverse transcriptase that catalyzes the addition of hexameric TTAGGG repeats to telomeres, the ends of chromosomal DNA [20]. The enzyme consists of three major components: telomerase reverse transcriptase (hTERT), telomerase-associated protein (TEP1), and telomerase RNA (TERC) [21-23]. Telomerase activation is essential for maintaining the telomere length and is required for cellular immortality. It has attracted substantial attention because telomerase activity has been observed in most types of human tumors, but not in adjacent normal cells [24-26]. In ~90% of advanced malignancy cases, high telomerase activity has been detected, which correlates well with increasing steady-state mRNA level of human telomerase reverse transcriptase [27].

Since inhibiting mTOR activity leads to cancer cell death while high levels of telomerase activity is associated with cancer cell proliferation, it is possible that mTOR may directly or indirectly regulate telomerase activity. However, there is no report regarding the role of chemotherapeutic agents on mTOR and its role in regulating the expression profiles of hTERT. In the current study, we investigate the changes in telomerase activity and mTOR activity after HCC cells are treated with 5-fluorouracil (5-Fu) and rapamycin. Our results suggest that 5-Fu treatment of HCC cell line SMMC-7721 could down-regulate both mTOR and telomerase activity, and inhibiting mTOR leads to further down-regulation of telomerase activity at the post-transcriptional levels.

## Methods

### Cell Culture

SMMC-7721 cells (a human hepatocarcinoma cell line) were maintained in Dulbecco's modified essential medium (DMEM) containing 10% fetal bovine serum (FBS). The cells were incubated at 37°C and 5% CO_2_. The growth media was changed every 2–3 days.

### Cell growth Assay

SMMC-7721 cells were suspended at a concentration of 5 × 104/ml, and then 200 μl of the cell suspension was placed in each well of a replicate 96-well microtiter plate. The cells were allowed to adhere overnight. Different concentrations (25 μg/ml, 75 μg/ml, 125 μg/ml, 137.5 μg/ml) of 5-Fu were added to the culture. MTT (Thiazolyl blue) assay was performed after 48 h. Ten microlitters of 5 mg/ml of MTT was added to each well followed by incubation for 4 h at 37°C. The formazan crystals were dissolved in 200 μl of DMSO. Optical density values (OD) were determined at a wavelength 570 nm. Each assay was performed three times and the average results were calculated.

### TUNEL Assay

Terminal deoxynucleotidyl transferase-mediated deoxyuridine nick end-labeling (TUNEL) staining was performed on SMMC-7721 cells to detect apoptotic cells. We used a commercial kit from ONCOGENE (Carpinteria, CA, USA). Accordingly, SMMC-7721 cells were fixed with 10% formalin, and slides with attached cells stained by TUNEL according to manufacturer's instructions, where the 3'-OH ends of fragmented nucleosomal DNA of apoptotic cells were specifically labeled using exogenous terminal transferase and fluorescently-labeled dNTP, then detected with a fluorescein labeled antibody specific to deoxyuridine. Slides with attached cells were then examined by fluorescence microscopy for positive staining.

### Plasmid and transient transfections

pBTdel-130 plasmid DNA containing 135 bp hTERT core promoter gene and Firefly luciferase was a gift from Dr. Jiyue Zhu. We used dual-luciferase reporter system, where the Renilla luciferase vector provides an internal control that serves as the baseline response. Transient transfections of SMMC-7721 cells were performed in 24-wells plates using Lipofectamin 2000 from Invitrogen according to the manufacturer instructions. SMMC-7721 cells were cultured in 24-well plates until they reached 85–90% confluence. 1 μl of Lipofectamin 2000 reagent and 0.4 μg pBTdel-130 plasmid DNA were used to transfect each well of cells in the absence of serum. After 4–6 h, the medium were replaced with 10% FBS DMEM. Approximately 24 h after the beginning of the transfection, the cells were exposed to 5-Fu and, or rapamycin. The cells luciferase activity was then analyzed by Dual-Luciferase Reporter Assay System.

### Telomerase activity assay

The telomerase activity was detected by the telomeric repeat amplication protocol and silver staining assay (TRAP-silver staining assay). Cells were washed once in phosphated-buffered saline and 10^5 ^cells were resuspended in 200 μl of 1 × CHAPS lysis buffer. After 30-minute incubation on ice, the suspension was centrifuged at 12 000 × g for 20 min at 4°C. Protein concentration was determined and extracts were stored at -80°C until assayed. A PCR-based telomerase assay and silver staining assay were performed according to a published protocol [28].

### RT-PCR for hTERT

Expression of hTERT mRNA was analyzed by RT-PCR amplification. Total RNA was isolated using Trizol (Invitrogen) according to the manufacturer's protocol. One μg of total RNA was reverse transcribed at 37°C for 45 min in the presence of random hexamer and Moloney murine leukemia virus reverse transcriptase (Gibco-BRL). Analysis of the expression of the telomerase subunit was performed by RT-PCR amplification. A 145-bp hTERT fragment was amplified using the primer pair 5'-CGGAAGAGTGTCTGGAGCAA-3' and 5'-GGATGAAGCGGAGTCTGGA-3'.

### Western blot analysis

Cells were lysed in 1 × SDS loading buffer and the lysates were centrifuged at 13,000 × g at 4°C for 30 min. Protein content in the supernatants was determined with the BCA Protein Assay system. Protein (30 μg) in cell extracts was resolved by 10% SDS-PAGE and transferred to PVDF membranes. After blocking with 5% nonfat dry milk in PBS containing 0.2% Tween 20, the membranes were incubated at 4°C overnight with antibodies. Blots were then incubated for 2 h at room temperature with secondary antibodies and then analyzed with the ELC chemiluminiscence substrate system (Amersham Biosciences, Piscataway, NJ). Antibodies specific for hTERT (Santa Cruz Biotechnology) and p70S6k Thr 389(Cell Signaling Technology) were used.

### Cell Proliferation Assay

SMMC-7721 cells (1 × 10^4^) were plated in triplicate onto 6-well culture plates in regular medium. The next day, the medium was changed, and rapamycin (10 nM 1 hr piror to 5-Fu) and 5-Fu was added. Cells were incubated for 36 hrs. Cells counts were performed with a hemocytometer.

### Statistical analysis

Differences in luciferase activity between 5-Fu treatment and control group were analyzed by Student's *t*-test. The probability (*P*) of 0.05 was considered to be significant.

## Results

### Inhibition of human hepatocarcinoma cells growth by 5-Fu is due to increased apoptosis

**Figure 1:**
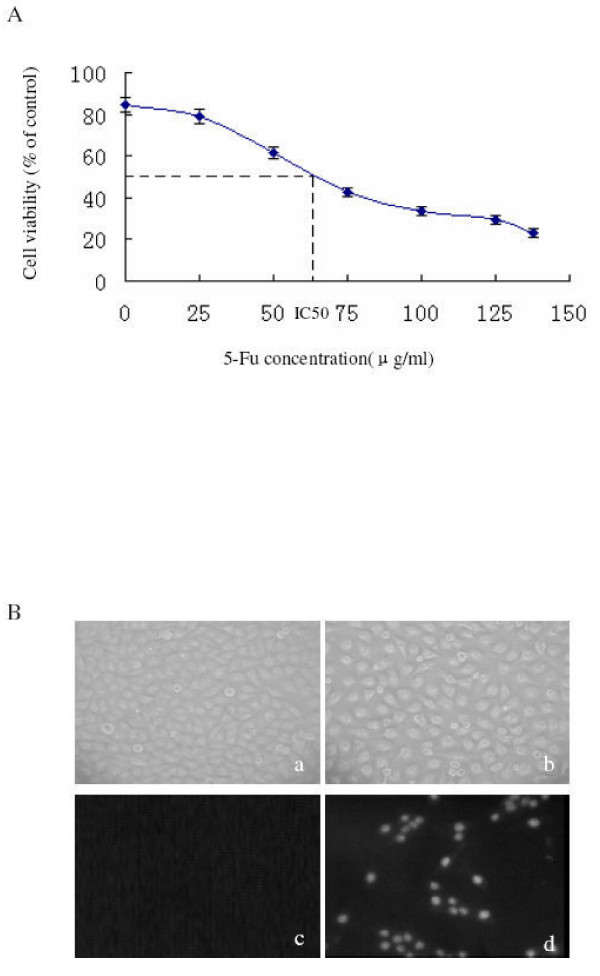
**Inhibition of human hepatocarcinoma cells growth by 5-Fu is due to increased apoptosis**. (A) SMMC-7721 cells were treated with different concentrations of 5-Fu for 36 h, and cell viability was measured by MTT assay. (B) SMMC-7721 cells were incubated with (b,d) or without (a,c) 5-Fu (62.5 μg/ml) for 36 h were stained by the TUNEL method to reveal DNA strand breaks indicative of apoptosis. (a) (b) bright field micrographs corresponding to (c) and (d), respectively. (c)(d), micrographs taken with fluorescent microscopy (400×).

SMMC-7721 cells were treated with different concentrations of 5-Fu for 36 h and cell viability were determined by MTT assay (Figure 1A). Cell viability was reduced under 5-Fu treatment in a dose-dependent manner. At the highest concentration (137.5 μg/ml) used, the inhibition on cell viability was 78.6%. The IC_50 _of 5-Fu was determined as 62.5 μg/ml.

Then SMMC-7721 cells were treated with the IC_50 _of 5-Fu (62.5 μg/ml) for 36 h, and the TUNEL staining technique was used to reveal DNA strand breaks, characteristic of cell apoptosis (Figure 1B). A significant numbers of cells (37.56%~45.32% at this concentration, based on triplicate experiments) underwent apoptosis after 5-Fu treatment, while few, if any, apoptotic cells were observed in the untreated samples.

These results indicated that 5-Fu could reduce the viability of SMMC-7721 cells in a dose-dependent manner, likely due to significant levels of cell death.

### Down-regulation of telomerase activity by 5-Fu at the transcriptional level

**Figure 2:**
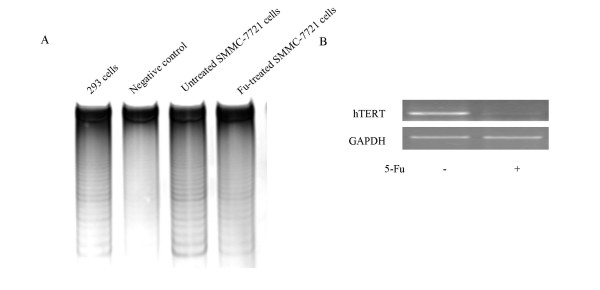
**Down-regulation of telomerase activity by 5-Fu**. (A) Telomerase activity assay. SMMC-7721 cells were harvested after incubation with 62.5 μg/ml of 5-Fu for 36 h. The telomerase activity was measured by TRAP-silver staining assay. (Lanes 1–4: 293 cell lysate as positive control, heat-treated (65°C, 10 min) SMMC-7721 cell lysate as negative control, SMMC-7721 cell lysate not treated with 5-Fu, SMMC-7721 cell lysate treated with 5-Fu (62.5 μg/ml) for 36 h,respectively). (B) hTERT mRNA expression determined with RT-PCR.

To investigate the effect of 5-Fu on telomerase activity in SMMC-7721 cells, telomerase activity was measured by TRAP-silver staining assay on cells harvested after 36 h treatment with 5-Fu at 62.5 μg/ml. Telomerase activity (Figure 2A), as well as the hTERT mRNA level (Figure 2B) were significantly reduced by 5-Fu treatment.

To confirm that down-regulation of telomerase activity by 5-Fu treatment occurs primarily at the transcriptional level, a plasmid carrying a firefly luciferase reporter gene driven by the 135 bp hTERT core promoter was transfected into SMMC-7721 cells and cells were cultured with or without 5-Fu treatment. The hTERT promoter activity was drastically reduced by 5-Fu treatment, indicating that in 5-Fu induced apoptosis of HCC cells, telomerase activity was down-regulated due to reduced hTERT expression at the transcription level.

### Inhibition of mTOR activity by 5-Fu

**Figure 3:**
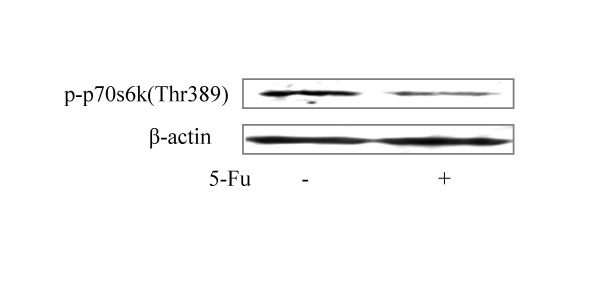
**Inhibition of mTOR activity by 5-Fu**. SMMC-7721 cells were treated with or without 5-Fu (62.5 μg/ml) for 36 h. Expression of P-p70S6K protein was detected by Western Blot. Cell lysates were immunoblotted with P-p70S6K Thr 389 antibody.

PI3K/Akt/mTOR signal pathway plays an important role in the course of initiation and progression of carcinoma. To study the role of mTOR in 5-Fu induced apoptosis of HCC cells, SMMC-7721 cells were treated with or without 5-Fu at the concentration of 62.5 μg/ml for 36 h, and then cell lysates were prepared, and Western analyses were performed with an antibody specific for the phosphotylated form of p70s6 kinase at Thr389, the position known to be phosphotylated by mTOR. The amount of Thr389 phosphotylated p70s6 kinase in the 5-Fu-exposed cells was much less than that in the untreated cells (Figure 3), indicating that 5-Fu down-regulated mTOR activity.

**Figure 4:**
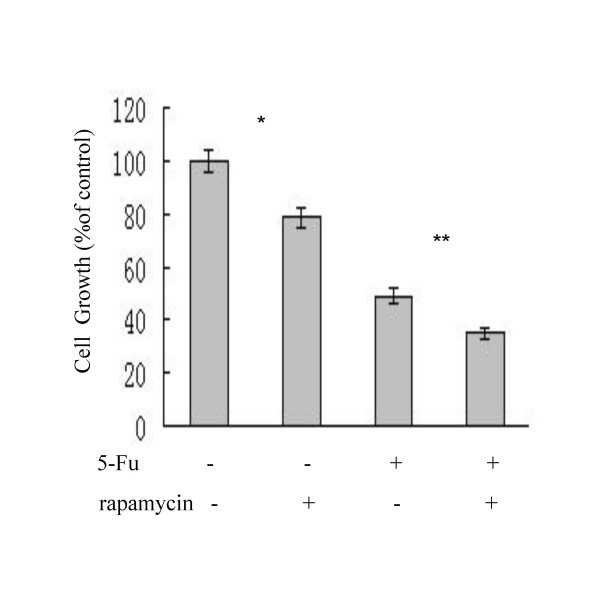
**Rapamycin and 5-Fu synergistically inhibit proliferation of SMMC-7721 cells**. SMMC-7721 cells were cultured in the presence of rapamycin (10 nM), or 5-Fu (62.5 μg/ml), or both drugs (10 nM rapamycin 1 h piror to 5-Fu) for 36 h. Cells counts were performed with a hemocytometer. The cell numbers were normalized to the untreated control. The results are shown as the mean ± SD of triplicate samples and are representative of two independent experiments. Statistical significance shown as * and ** was determined by t test, with p > 0.05 defined as significant.

To investigate the role of mTOR in 5-Fu induced apoptosis of HCC cells, rapamycin, a specific inhibitor of mTOR, was used to block the PI3K/Akt/mTOR pathway. SMMC-7721 cells were cultured in the presence of rapamycin (10 nM 1 hr piror to 5-Fu) or 5-Fu (62.5 μg/ml) or both for 36 h. As shown in Figure 4, at the concentration tested, rapamycin alone reduced cell growth rate by ~20%, 5-Fu alone reduced cell growth rate by ~50% and the two drugs together reduced cell growth rate by nearly 70%, demonstrating synergistic or additive effect of 5-Fu and rapamycin in inhibiting growth of HCC cells in vitro.

### Rapamycin reduces hTERT protein expression in synergy with 5-Fu

**Figure 5:**
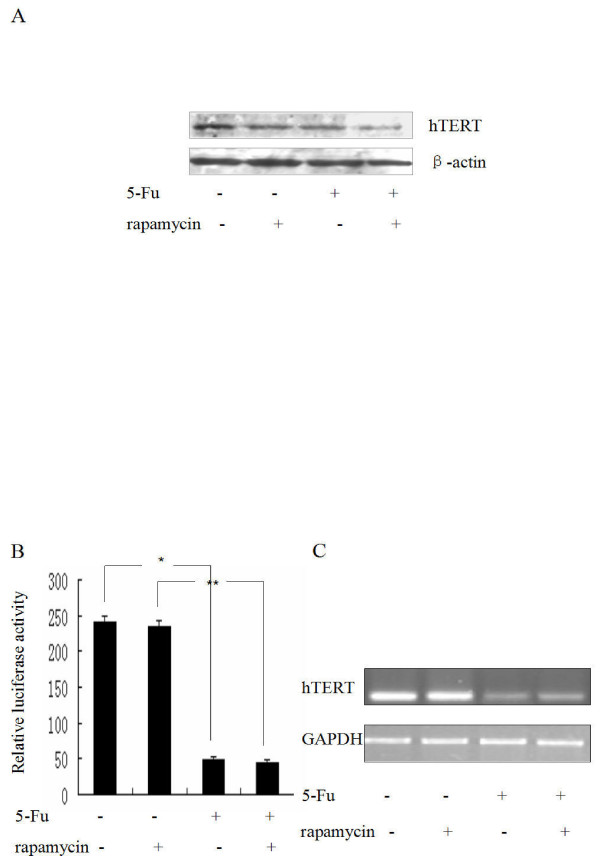
**Rapamycin did not reduce hTERT transcription but reduced hTERT protein expression**. (A) The effect of rapamycin on hTERT protein expression was detected by Western Blot. SMMC-7721 cells were treated with rapamycin, or 5-Fu or both for 36 h, cell lysates were immunoblotted with hTERT antibody. (B) The effect of rapamycin and 5-Fu on hTERT promoter activity. SMMC-7721 cells were transient transfected with pBTdel-130 plasmid DNA, which contains the luciferase reporter driven by the core hTERT promoter, whole cell extract was then prepared from the cells and luciferase activity was measured by Dual-Luciferase Reporter Assay System (lands are as labeled on the figure). The mean ± SD of four experiments is shown; statistical significance shown as * and ** was determined by t test, with p > 0.05 defined as significant. (C) hTERT mRNA expression determined with RT-PCR.

To investigate the link between mTOR and telomerase activity in 5-Fu induced apoptosis of HCC cells, the effect of rapamycin on hTERT mRNA transcription and protein expression was examined. Treatment of SMMC-7721 cells with either rapamycin or 5-Fu alone resulted in significant reduction of hTERT protein expression compared with that of the untreated cells, and dual treatment caused a more profound reduction in the hTERT protein level than either single drug treatment (Figure 5A), suggesting a synergistic/additive effect of the two drugs on reducing the protein level of hTERT.

To investigate the underlying mechanism, the hTERT promoter activity and hTERT mRNA levels were analyzed in the cells treated with or without both drugs. SMMC-7721 cells transiently tranfected with the reporter plasmid were treated with rapamycin (10 nM) or 5-Fu (62.5 μg/ml) or both drugs (10 nM rapamycin 1 h piror to 5-Fu) for 36 h. Again, 5-Fu nearly abolished the activity of the hTERT promoter (Figure 5B) or mRNA expression (Figure 5C). On the other hand, rapamycin treatment had no effect at all on hTERT promoter activity and hTERT mRNA level, indicating that rapamycin probably reduced hTERT protein expression at the post-transcriptional level.

## Discussion

The reactivation of telomerase activity is a vital step in tumorigenesis. Beyond its role in telomere maintenance, telomerase provides additional functions in DNA repair and cell survival. Telomerase protects cells from apoptosis and necrosis, and stimulates growth under adverse conditions [29]. Inhibition of telomerase activity in cancer cells is a potent factor in the abrogation of cellular immortalization. A number of different approaches have been developed to inhibit telomerase activity in human cancer cells. Different components and types of inhibitors targeting various regulatory levels have been regarded as useful for telomerase inhibition. Many telomerase inhibitors seem to be most efficient when combined with conventional chemotherapeutics. It has been demonstrated that telomerase may be involved in triggering apoptosis, but the underlying molecular mechanism remains unclear [30]. In the present study, we showed that 5-Fu treatment of SMMC-7721 cells induces apoptosis and telomerase activity down-regulation within 36 h. Moreover, this is accompanied by reduction of hTERT mRNA and hTERT protein.

In principle, survival signals are ideal targets for anticancer therapeutic strategies because blocking these signals leads to the death of cells that are dependent upon them. Increasing evidences implicate mTOR as a central player in cell proliferation, migration, and survival [31-33]. The mTOR protein is involved in the regulation of cyclins D1/A, cyclin-dependent kinases, cyclin-dependent kinase inhibitors (p21Cip1 and p27Kip1), retinoblastoma protein, RNA polymerase I/II/III-transcription and translation [34-37]. Suppression of these mTOR-mediated survival signals provides the opportunity to reactivate default apoptotic pathways in cancer cells and allow them to proceed on the path of death [38]. Three potent and specific mTOR inhibitors have been reported which are either rapamycin or rapamycin derivatives: rapamycin, CCI-779 (also called cell-cycle inhibitor-779, rapamycin-42,2,2-bis(hydroxymethyl)-propionic acid; Wyeth-Ayerst, PA, USA) and RAD001 (also called everolimus or 40-O-(2-hydroxyethyl)-rapamycin; Novartis AG, Basel, Switzerland). In addition to being a fungicide and immunosuppressant, rapamycin has also been proposed as a potential therapeutic for cancer treatment and for restenosis prevention [39,40].

Our present study attempts to link these two important factors of human tumorigenesis in hepatocellular carcinoma (HCC), which is one of the leading causes of cancer and cancer-related death in China, in the context of conventional chemotherapeutics (i.e. 5-Fu) induced apoptosis of HCC cells. We have demonstrated that in 5-Fu induced apoptosis of HCC cells SMMC-7721 in a dose-dependent fashion, and 5-Fu reduced telomerase activity of these cells primarily through reduction in hTERT transcription. The 5-Fu treatment also reduced mTOR activity. Inhibition of mTOR activity with its specific inhibitor rapamycin resulted in decrease of SMMC-7721 cells viability and down-regulation of hTERT protein expression, although rapamycin did not affect hTERT transcription. The two effects, reduction of cell viability and down-regulation of hTERT protein, caused by 5-Fu and rapamycin were synergistic or additive and that this would be a potential chemotherapeutic combination for hepatocellular cancer. Our findings indicated that mTOR signal molecule may be involved in the regulation of telomerase activity at post-transcriptional level. Phosphatidylinositol-3-kinase (PI3-K) pathway has been reported as an important intracellular mediator frequently activated in cancer cells. PI3K activates a number of signaling molecules, such as Akt. Recent studies show mTOR is an important substrate of Akt. The down-regulation of mTOR activity may be through the inhibition of PI3K/Akt signal pathway.

Thus, 5-Fu functions to induce cell death by regulating telomerase activity through two distinct mechanisms: 1) direct effect at the transcriptional level and 2) indirectly by down-regulating mTOR, which leads to reduced telomerase protein level. Thus, our results suggest that mTOR may be a likely chemotherapeutic target for cancer.

## Conclusion

Chemotherapeutic agent, 5-Fu, down-regulated telomerase activity at both transcriptional level and PI3K/Akt/mTOR pathway-dependent post-transcriptional level to facilitate hepatocellular carcinoma cell apoptosis.

## Abbreviations

hTERT, human telomerase reverse transcriptase; PI3K, phosphatidylinositol-3-kinase; mTOR, mammalian target of rapamycin; 5-Fu, 5-fluorouracil.

## Competing interests

The author(s) declare that they have no competing interests.

## Authors' contributions

XB carried out the molecular genetic studies and drafted the manuscript.

FJ and WW carried out the immunoassays. XG participated in the statistical analysis. MW and LW conceived of the study, and participate in its design and coordination. All authors read and approved the final manuscript.

## Pre-publication history

The pre-publication history for this paper can be accessed here:


